# Development of Porous MoO_2_ Pellet Target for ^99^Mo/^99m^Tc Generator

**DOI:** 10.3390/ma16206713

**Published:** 2023-10-16

**Authors:** Xiangrong Hu, Tatsuya Suzuki

**Affiliations:** Department of Nuclear Technology, Nagaoka University of Technology, Nagaoka, Niigata 940-2188, Japan; xiangrong_hu@outlook.com

**Keywords:** MoO_2_, sinter, (n,γ) reaction target, porous materials

## Abstract

Technetium-99m(^99m^Tc) is used worldwide in 85% of nuclear medicine diagnostic imaging procedures. We developed porous MoO_2_ pellets as an alternative to reactor-based targets in an (n,γ) reaction for producing Technetium-99m (^99m^Tc) in nuclear medicine. The pellets, formed through a manufacturing process involving mixing, sintering, eluting, and drying, offer advantages such as selective dissolution and improved yield. This research offers a potential solution for stable ^99m^Tc production, focusing on porous molybdenum dioxide (MoO_2_) as a target material due to its insolubility in water. Using potassium molybdate (K_2_MoO_4_) as a pore former, we developed porous MoO_2_ pellets that facilitate efficient technetium extraction and target recycling. This approach offers control over pore formation and shows promise in addressing supply challenges and enhancing ^99m^Tc production.

## 1. Introduction

Technetium-99m (^99m^Tc) is used in 85% of nuclear medicine diagnostic imaging procedures worldwide [1,2,3,4]; almost all ^99m^Tc used for this purpose is obtained from the radioactive decay of molybdenum-99 (^99^Mo), which is produced by processing irradiated highly enriched uranium (HEU) targets [5]. The use of HEU raises concerns from a proliferation perspective. Furthermore, the reactors used for the production of ^99^Mo are susceptible to uncertainty due to planned and unplanned shutdowns and decommission [6]. The increasing demand for ^99m^Tc has prompted efforts to solve the supply shortages. Under such circumstances, several alternative methods have been proposed, such as reactor-based solutions, accelerator-based solutions, and the conversion of HEU to LEU (low-enriched uranium) [7,8,9]. The use of HEU also has a problem with plutonium generation, which makes its use difficult for signatory countries of the NPT, such as Japan. ^98^Mo used to produce ^99^Mo by the (n,γ) reaction was proposed as a promising solution, which has inspired further development, with the advantages of radioactive waste, cost reduction, and non-proliferation [10]. The neutron activation of ^98^Mo by the reaction (^98^Mo(n,γ)^99^Mo) has a small activation cross-section (0.13 b for thermal neutrons) compared with the neutron fission production of ^99^Mo (584 b for thermal neutrons) [11]. From this perspective, it is required to increase the yield of ^99^Mo, and a variety of research has been carried out towards this objective (e.g., increasing the enrichment of ^98^Mo, and increasing the density of the pellet) [12,13].

The common practice is to use MoO_3_ targets of natural abundance, which is economically preferred [8]. However, the Mo/Tc ratio in MoO_3_ is usually high, which makes it difficult to use conventional generators to extract ^99m^Tc. To overcome this issue, the ^99^Mo is separated from the MoO_3_ target by dissolving it in an alkali solution and passing it through an adsorbent material in a column [14]. Because of the advantage of increased ^99^Mo production yield, the use of ^98^Mo target material with enrichment of >95% has also drawn wide attention [5]. Meanwhile, the relatively high cost of highly enriched ^98^Mo requires more effort to recover the unutilized ^98^Mo of the target. The crystalline density of MoO_3_ is about 4.7 g/cm^3^; MoO_3_ has limited solubility in water (1 g/L at 20 °C) and can be easily dissolved in sodium hydroxide (NaOH). The obtained solution can be further processed by separation techniques (e.g., column chromatography and solvent extraction). Mo metal is another typically used target, which can be dissolved in alkaline hydrogen peroxide (H_2_O_2_) or electrochemically. However, another oxide, molybdenum dioxide (MoO_2_), has been comparatively much less explored as a target.

MoO_2_ is almost insoluble in water [15,16] and contains more ^98^Mo per volume compared with MoO_3_. This insolubility provides the possibility of processing the irradiated target by water, rather than dissolving the entire quantity of molybdenum oxide in the alkali solution. This technique proposes a porous structure and selectively dissolves only the ^99m^Tc located on the surface. The solution can be further handled, and the undissolved target can be recovered. This process takes advantage of the large surface and the short diffusion distance from the inside of the solid to the surface, which allows for efficient extraction of the ^99m^Tc. On the other hand, MoO_2_ is less soluble than MoO_3_, which means that it is more likely to be adsorbed onto the column and less likely to be eluted with the Tc-99m. This can reduce the Mo/Tc ratio in the generator and increase the yield and quality of the Tc-99m, which is of benefit to the separation operation of molybdenum–technetium and the recycling of the molybdenum target. 

Porous oxide materials have garnered significant attention in various scientific and industrial applications due to their properties, such as high surface area and a tunable pore size [17]. One widely explored method for the fabrication of these materials involves the use of pore formers [18]. Pore formers, also known as sacrificial templates, porogens, or space holders, are materials intentionally added during the fabrication of porous materials to create voids or pores within the final structure [19]. These pores can range from nanometers to micrometers in size and play a crucial role in determining the material’s properties and applications. The advantages of using space holders in porous materials are manifold. They allow precise control over pore size, shape, and distribution, enabling tailored materials for specific applications like catalysis adsorption and drug delivery [20]. Furthermore, these methods can enhance the overall surface area and porosity of the oxide, which can improve the material’s reactivity and performance. 

In this study, potassium molybdate (K_2_MoO_4_) is used as the space holder in the pore-forming process. The selection of K_2_MoO_4_ as a porogen is driven by its exceptional attributes, which make it a compelling choice for creating well-defined pores in MoO_2_ pellets, all while preserving their structural integrity and chemical composition. One of its standout features is its relatively high melting point, which is compatible with MoO_2_, the core material of interest. Moreover, the introduction of only the element potassium as a new component brings a distinctive aspect to the composition, contributing to the material’s unique properties. Additionally, there is a significant contrast in solubility in water when compared to MoO_2_, further enhancing its effectiveness as a porogen in the pore-forming process. These combined characteristics make K_2_MoO_4_ a standout candidate for our study, ensuring precise control over pore formation while maintaining the integrity of the resulting oxide material.

Hence, we propose to develop a porous MoO_2_ pellet target as a complement to the (n,γ) reaction target selection. 

In our previous work, we conducted exploratory experiments to select the appropriate pore former and determine the sintering conditions. Building on this foundation, the current study focuses on the utilization of MoO_2_ powder mixed with potassium molybdate (K_2_MoO_4_), which offers several advantageous properties, as mentioned above. Notably, the melting point of MoO_2_ is 1100 °C [21], while K_2_MoO_4_ exhibits a lower melting point of 919 °C. Furthermore, K_2_MoO_4_ demonstrates excellent solubility, which is 164.5 g/100 g H_2_O at 25 °C. 

The fabrication involved the process of sintering, followed by the formation of pores through dissolving the pore formers. Subsequently, we subjected the porous pellets to thorough characterization.

## 2. Materials and Methods

### 2.1. Manufacture of Porous MoO_2_ Pellet

The molybdenum(Ⅳ) oxide (Strem Chemicals, 99%) powders were mixed with potassium molybdate (FUJIFILM Wako Pure Chemical Industries, Ltd., Osaka, Japan); zinc stearate (FUJIFILM Wako Pure Chemical Corporation, 12.0~14.5%) was added as a lubricant for sintering, which decomposed after the sintering temperature reached 300~400 °C. The mixed compounds were weighted (Azpro Analytical Balance 120g ASR124/E, Minimum measurement: 0.0001 g) to reach the expected ratio (70% MoO_2_ and 30% K_2_MoO_4_), and weighted powders were ground using the agate mortar and pestle since the fine powders can benefit the sinter. Furthermore, the ground powders were filled into the pressing die (Φ7 mm, δ1 mm), by which the pellet form was obtained. The dark and dry pellet was sintered using a tube furnace (TMF-300N/-500N/-700N, AS ONE Corporation, Osaka, Japan). 

In addition, by taking advantage of the extreme insolubility of molybdenum(Ⅳ) oxide in water, a compound that is easily soluble in water can be taken for consideration as a pore-forming compound. The selection of the pore former requires a higher solubility in water and is unreactive with MoO_2_, while potassium has an extremely high solubility in water, which is 164.5 g in 100 g water at 25 °C. Simultaneously, the pore former also requires a high melting point, so that they can be sintered under high temperatures. The melting point of MoO_2_ is 1100 °C and 919 °C for K_2_MoO_4_. The sinter was carried out under 900 °C for 6 h, which is below the melting point of the smaller pellet composition.

The sintered pellet was eluted with 100 mL ultrapure water, by which the K_2_MoO_4_ can be easily dissolved, and the pores were formed. The eluted pellet was dried under a vacuum.

The manufacturing process of porous MoO_2_ pellets is as follows:

- Mixing of MoO_2_ and pore former powders.
- Grinding to fine powders.
- Pressing to form a pellet.
- Sintering.
- Eluting and drying.

In the first two stages, MoO_2_ was combined with K_2_MoO_4_ powders, and the mixture was finely ground before being compressed to form the desired structure. These initial steps were crucial to enhance the sintering process.

For the sintering process itself, the temperature profile was carefully controlled. Initially, the temperature was set to 400 °C and maintained for 1 h to facilitate the decomposition of zinc stearate, a component within the mixture. Following this, the temperature was raised to 900 °C and held for 6 h. This stage promoted grain growth and facilitated the binding of particles to one another.

In the final step, due to the disparity in solubility between MoO_2_ and K_2_MoO_4_, K_2_MoO_4_ was selectively dissolved into water, leaving behind pores within the MoO_2_ pellet.

In these steps, precise control over the pore system can be achieved by adjusting the grinding time, altering the ratio of mixtures, or fine-tuning the sintering conditions. This level of control allows us to tailor the resulting pore structure to meet the specific requirements and desired characteristics for our application.

### 2.2. Characterization of Porous MoO_2_ Pellet

A micrometer, a caliper, and an electric balance were used to physically measure the obtained pellet. The X-ray diffraction (XRD) apparatus (RINT-2500HF+/PC, Rigaku Corporation) and scanning electron microscope–energy dispersive spectroscopy (SEM-EDS, JEOL JCM-6000Plus/EDS, Melville, NY, USA) were utilized for characterization. 

## 3. Results

### 3.1. Obtained Pellet

**Figure 1:**
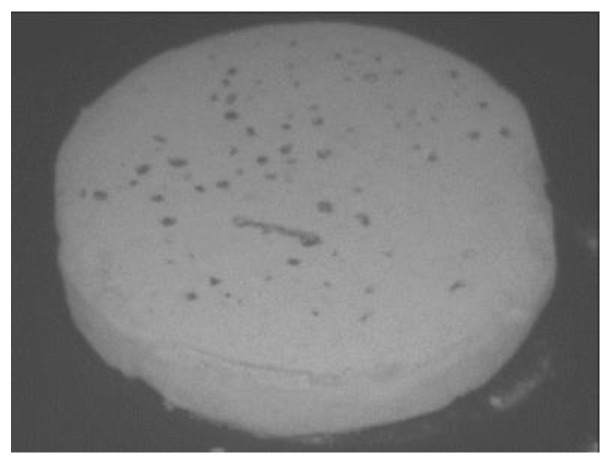
The obtained MoO_2_ pellet.

The obtained pellet is composed of a combination of compounds, with approximately 70% MoO_2_ and 30% K_2_MoO_4_ by volume. This unique composition contributes to its distinctive properties. The pellet itself possesses specific dimensions, featuring a diameter of 7.0 mm and a thickness of 1.1 mm, which were carefully controlled during the manufacturing process. In Figure 1, the overall structure of the porous pellet is visually depicted using scanning electron microscopy (SEM) at a 30× magnification level. This image reveals the intricate pore network within the pellet, and the pores are readily discernible through the SEM, highlighting the achievement of the desired porous structure.

### 3.2. Structural and Phase Analysis of the Porous Material

**Figure 2:**
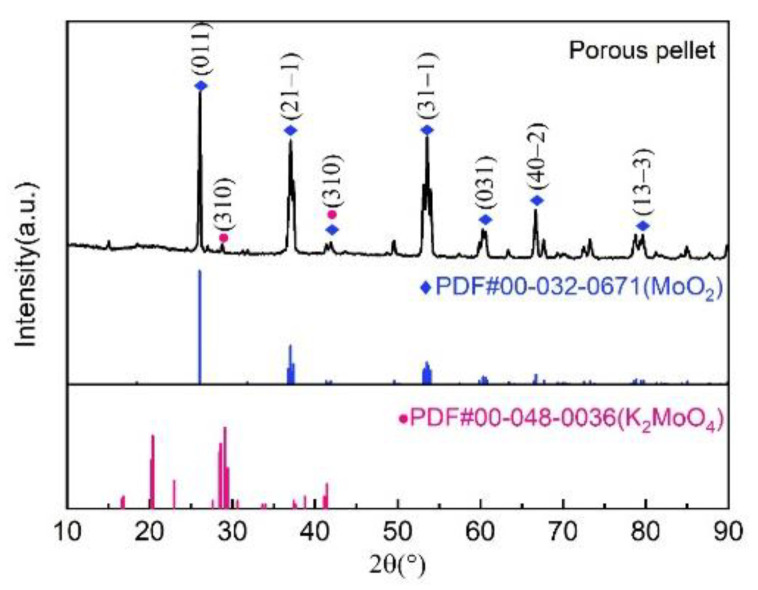
The characterization of XRD for MoO_2_ porous pellet.

The XRD results of sintered and eluted MoO_2_ pellets are shown in Figure 2. From the porous MoO_2_ pellet XRD pattern, the peak positions and relative intensities well matched those of the MoO_2_ PDF card, which suggests that MoO_2_ is a significant component in the sample. Furthermore, the peak positions that matched with the K_2_MoO_4_ PDF card had low intensity. This result indicates that the target mostly remained MoO_2_, and the pore former was essentially removed. A high degree of purity is indicated, since the pattern closely matched the reference MoO_2_ crystalline phase.

### 3.3. Morphological Analysis of the Porous Material

**Figure 3:**
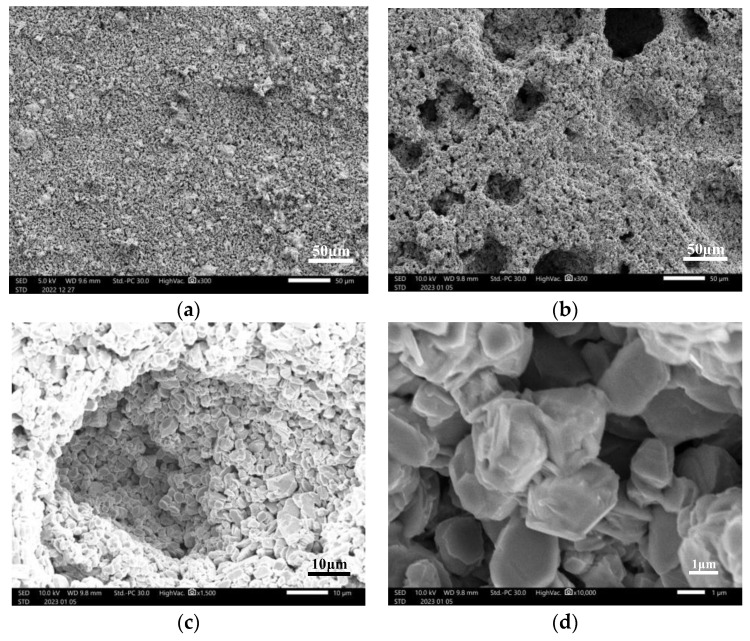
Microscopic images of the pellet observed in SEM before and after pore formation: (**a**) pellet before elution at 300 magnifications; (**b**) pellet after elution at 300 magnifications; (**c**) pore observed at 1500 magnifications; and (**d**) pore observed at 10,000 magnifications.

The SEM results of the MoO_2_ pellet are shown in Figure 3. Comparing the SEM pattern of MoO_2_ pellet before and after pore formation, the pores are well observed by SEM. This result indicates that the pores were well formed after the pellet was eluted in ultrapure water.

The SEM figure unveils a heterogeneous pore system within the material, featuring both macropores and micropores. Macropores, circular in shape with an average size of 30 μm (um), provide substantial voids for large-scale interactions, while the hexagonal voids of micropores, measuring under 1 micrometer (um), contribute to a finely structured and organized porosity. This intricate combination of macropores and micropores presents a diverse and versatile landscape of voids within the material, potentially influencing various physical and chemical processes.

## 4. Discussion

Throughout the experiment, extensive efforts were devoted to identifying suitable pore formers and optimizing sintering conditions. Ultimately, the production of a porous MoO_2_ target was successfully accomplished by sintering at 900 °C using a pore former, followed by elution with ultra-pure water. This sintering temperature aligns closely with the ideal sintering temperature for MoO_2_, which reaches 81.8% of the melting point of MoO_2_. In the pellet fabrication process, MoO_2_ and K_2_MoO_4_ are added in a ratio of 7:3. When considering the relative density, the calculated quantities for producing a pellet with a 7 mm diameter and 1 mm thickness were 0.1220 g of molybdenum dioxide and 0.0354 g of potassium molybdate. The resulting sintered target exhibited dimensions of 7.0 mm in diameter and 1.1 mm in thickness. The weight of the target obtained after the elution process with water to form pores and drying in a vacuum was 0.1214 g. Remarkably, this weight closely matches that of the initially added molybdenum dioxide. This outcome demonstrates that during the pore formation process, potassium molybdate primarily dissolves in ultra-pure water, while molybdenum dioxide remains in the pellet due to its extremely insolubility in water.

Our findings were further validated through X-ray diffraction (XRD) and scanning electron microscope (SEM) analyses. The XRD results revealed that the peak positions of the obtained sample are well matched to those of the molybdenum dioxide PDF card, and that only a small part of the peaks corresponded to the potassium molybdate PDF card; these peaks exhibited low intensity, which means potassium molybdate was well removed. SEM imaging effectively demonstrated significant structural alterations in the pellet both before and after the elution process. After the elution, pores became conspicuously apparent, thereby affirming their formation because of the dissolution of potassium molybdate. These pores play a critical role in expanding the material’s surface area and reducing diffusion distances. However, it is worth noting that an excessive formation of pores carries the risk of compromising the material’s structural integrity.

The utilization of porous MoO_2_ pellets presents several notable advantages, as evidenced by the experimental results. These advantages encompass enhanced selective dissolution, improved generator yield, efficient technetium extraction, production stability, and target recyclability. These benefits collectively address supply challenges, elevate the quality of ^99m^Tc production, and provide a promising solution for stable and efficient production in nuclear medicine applications. The MoO_2_ targets with well-formed pores achieved through this method have great potential in molybdenum–technetium separation and target recycling for the stable and efficient production of ^99m^Tc.

## 5. Conclusions

The production of porous MoO_2_ pellets has been successfully achieved. We found suitable pore formers and sintering conditions, resulting in the creation of pellets with well-formed pores. The retained molybdenum dioxide within the pellets and the dissolution of potassium molybdate during the pore formation process were confirmed through weight measurements. XRD analysis revealed a close match to the molybdenum dioxide pattern, indicating the successful removal of potassium molybdate. Additionally, SEM images provided visual evidence of the presence of pores after elution. These findings unequivocally demonstrate the feasibility of creating porous MoO_2_ pellets, underscoring their potential for molybdenum–technetium separation and target recycling, thus offering a promising solution for the stable and efficient production of ^99m^Tc.

## Data Availability

Data is contained within the article.
